# The moderating role of avoidance behavior on anxiety over time: Is there a difference between social anxiety disorder and specific phobia?

**DOI:** 10.1371/journal.pone.0180298

**Published:** 2017-07-03

**Authors:** Myriam Rudaz, Thomas Ledermann, Jürgen Margraf, Eni S. Becker, Michelle G. Craske

**Affiliations:** 1 Department of Psychology, Utah State University, Logan, Utah, United States of America; 2 Department of Psychology, Ruhr-Universität Bochum, Bochum, Germany; 3 Behavioural Science Institute, Radboud University of Nijmegen, Nijmegen, Netherlands; 4 Department of Psychology, University of California Los Angeles, Los Angeles, California, United States of America; Shinshu University School of Medicine, JAPAN

## Abstract

Theories of anxiety disorders and phobias have ascribed a critical role to avoidance behavior in explaining the persistence of fear and anxiety, but knowledge about the role of avoidance behavior in the maintenance of anxiety in social anxiety disorder relative to specific phobia is lacking. This study examined the extent to which avoidance behavior moderates the relationship between general anxiety at baseline and 18 months later in women with a diagnosed social anxiety disorder (*n* = 91) and women with a diagnosed specific phobia (*n* = 130) at baseline. Circumscribed avoidance of social and specific situations were clinician-rated using the Anxiety Disorders Interview Schedule-Lifetime (ADIS-IV-L), and general anxiety was measured using the Beck Anxiety Inventory (BAI). Moderated regression analyses revealed that (a) general anxiety at baseline predicted general anxiety at follow-up in both women with a specific phobia and women with a social anxiety disorder and (b) avoidance behavior moderated this relationship in women with a specific phobia but not in women with a social anxiety disorder. Specifically, high avoidance behavior was found to amplify the effect between general anxiety at baseline and follow-up in specific phobia. Reasons for the absence of a similar moderating effect of avoidance behavior within social anxiety disorder are discussed.

## Introduction

Social anxiety disorder and specific phobia are the most common lifetime anxiety disorders with prevalence rates in the United States estimated to be as high as 10.7 and 15.6 percent, respectively [1]. For Europe, these estimates were in the range of 1.9 to 13.7 percent for social anxiety disorder and 7.7 to 15.3 percent for specific phobia [2, 3]. Social anxiety disorder frequently emerges in adolescence or early adulthood and specific phobia in childhood [4–6]. Both social anxiety disorder and specific phobia are commonly accompanied by other mental disorders, including mood disorders and other anxiety disorders [7, 8], and it has been repeatedly shown that women are significantly more likely to be affected than men [9, 10].

Social anxiety disorder involves a marked and persistent fear of negative evaluation in social and/or performance situations. The individual fears that he or she will act in a way or show anxiety symptoms that will be humiliating or embarrassing [11]. One of the most common reported fears is public speaking [12]. Specific phobia is characterized as a marked and persistent fear that is excessive or unreasonable, cued by the presence or anticipation of a specific object or situation [11]. Animal phobia has been found to be one of the most prevalent types [9]. In each of these disorders, avoidance behavior (or endurance of feared situations with dread) is a defining feature [11].

Several theories of phobias and anxiety disorders have assigned a critical role to avoidance behavior in explaining the persistence of fear and anxiety (for an overview see [13]). In accordance with *conditioning theory* [14, 15], fear persists because avoidance of the conditioned stimulus (CS) prevents the extinction that normally occurs through repeated non-reinforced exposures. From a *cognitive perspective*, avoidance behavior prevents the gathering of information that disconfirms catastrophic misappraisals, and consequently such misappraisals and their resultant fear and anxiety are maintained [16, 17]. Another cognitive model emphasizes the role of fear prediction, with *overprediction of fear* [18, 19] motivating avoidance behavior, which in turn prevents disconfirmatory opportunities, so that anxiety remains high. A third cognitive model emphasizes the *cognitive rehearsal of negative outcomes* [20, 21] that sometimes occurs with rumination or mental reflection about aversive events in the absence of direct or vicarious experience (i.e., avoidance), thereby leading to inflations of fear.

*Cognitive models of social anxiety disorder* posit that socially anxious individuals engage in various ‘in-situation or subtle safety behaviors’, such as avoiding eye contact or avoiding pauses while talking [22–24]. These safety behaviors prevent socially phobic individuals from processing exposures accurately because they attribute the non-occurrence of feared outcomes (e.g., embarrassment, rejection) to the safety behaviors in which they engaged and, thus, their unrealistic beliefs maintain. Also, safety behaviors may increase feared anxiety symptoms (e.g., holding a glass tightly may increase the likelihood of shaking) or may inhibit performance in such a way as to negatively influence the responses of others (e.g., remaining quiet in social conversations may increase the likelihood of being ignored by others) [25, 26].

The role of avoidance behavior is further implicated by exposure-based treatments for anxiety disorders and phobias that replace avoidance with approach toward phobic stimuli [27–31]. More specifically, exposure-based therapies have been found to be effective for specific phobias [32, 33] and superior to placebo or alternative treatment approaches, including relaxation, progressive muscle relaxation, tension techniques, and cognitive therapy [34]. Several meta-analyses also report the efficacy of exposure based treatments for social anxiety disorder, with some showing equivalent effects when exposure is conducted alone or in combination with cognitive restructuring [35–39] and one finding provides additional support for the combination with cognitive restructuring [40]. A number of studies also support the superior efficacy of in vivo exposure combined with a decrease in safety behaviors relative to exposure alone in social anxiety disorder [26, 41, 42]. Finally, a recent study emphasizes the treatment of socially anxious individual’s fixed beliefs about their anxiety in cognitive behavioral therapy [43].

Looking across anxiety disorders, evidence exist that individuals with social anxiety disorder had a poorer treatment response after cognitive-behavioral therapy than those with generalized anxiety disorder [30, 44] and posttraumatic stress disorder [30]. These findings may support the differentiation between distress or misery disorders (which include depressive disorders, generalized anxiety disorder, and posttraumatic stress disorder) and the fear disorders (which include panic disorder, agoraphobia, social anxiety disorder, and specific phobia) [45]. In contrast, it was found that a lack of positive affect appears to be strongly associated with both social anxiety disorder and depression, which is at odds with the separation of social anxiety disorder as a fear and depression as a distress or misery disorder [46].

Although there are a number of studies showing that exposure is effective in treating social anxiety disorder and specific phobia, the influence of avoidance behavior on the maintenance of anxiety over time has not been investigated directly in individuals with social anxiety disorder and individuals with specific phobia. The aim of the present study was to examine the extent to which avoidance behavior moderates the relationship between general anxiety at baseline and 18 months later in women with a DSM-IV social anxiety disorder and women with a DSM-IV specific phobia. Specifically, we tested two main hypotheses derived from conditioning [14, 15] and cognitive models [16–21]. The first hypothesis was that general anxiety at baseline and circumscribed avoidance behavior between baseline and follow-up would both predict general anxiety at follow-up in women with social anxiety disorder and specific phobia. The second hypothesis was that the relationship between general anxiety at baseline and follow-up would be moderated by circumscribed avoidance behavior. Because socially anxious individuals can easily show ‘in-situation or subtle safety behaviors’ that maintains their social anxiety [22–24], we expect a stronger moderating effect of avoidance for the specific phobia group as compared to the social anxiety disorder group. Specifically, we assumed that low avoidance behavior would have a buffering effect, whereas high avoidance behavior would amplify the strength of the relationship between general anxiety at baseline and follow-up.

## Method

### Participants

The data were derived from the Dresden Prediction Study (DPS), a prospective epidemiological study designed to collect data on the prevalence rates, incidence, course, and risk factors of mental disorders in young women (for details about the study see [47–50]). The focus of the DPS was to investigate women during early adulthood because of the high prevalence rates of anxiety disorders in women [51, 52]. The protocol of the DPS was approved by the local ethics board, the ‘sächsischer Datenschutz’. The sample was drawn randomly from the German Dresden government registry of residents in 1996. Of the 9,000 addresses received from the registry office, 5,204 women were located and eligible for the study. Of these, 3,065 women responded, resulting in a response rate of 58.9%. A subsample of 1,877 were willing to take part in the diagnostic interview and to complete a package of self-report measures at baseline and 1,396 at the follow-up survey approximately 18 months apart (see [48] for a flow diagram of the number of respondents and response rates at baseline and follow-up). The baseline interviews were conducted between July 1996 and September 1997, and the follow-up interviews between December 1997 and February 1999. Inclusion criteria were being female and being 18–25 years of age at the time of the baseline investigation. There were no significant differences in overall mental disorders at baseline between women with complete data at baseline and follow-up and women with missing interview or self-report data at follow-up (*t*(802) = -1.40, *p* = .161). The present study refers to 1,396 women, aged between 18 to 24 years at the time of sampling, who completed a diagnostic interview and self-report questionnaires at baseline and follow-up.

### Procedure and measures

The women received a letter with detailed information about the DPS and a reply card to provide written informed consent to participate in the study. The interested women were invited for the diagnostic interview conducted by trained clinical interviewers. In addition, a package of questionnaires was administered. Participants willing to join the second diagnostic interview were invited on average 18 months later. There were no financial reimbursements for participants in the study.

#### Diagnostic assessment

The diagnostic assessments were based on the German version of the Anxiety Disorders Interview Schedule-Lifetime (ADIS-IV-L) [53, 54]. The ADIS-IV-L is a structured interview for diagnosing Axis I disorders, including anxiety disorders, affective disorders, somatoform disorders, substance use disorders, eating disorders, and children’s disorders according to DSM-IV [55]. In the baseline interview, questions were asked about mental problems during the entire lifetime and during the last seven days (lifetime and point prevalence, respectively). The follow-up interviews referred to the mental problems that had occurred since the baseline interview and during the last seven days (period and point prevalence, respectively). In the current study, the seven-day point prevalence at baseline was used to compose the two groups, women with a baseline social anxiety disorder and women with a baseline specific phobia. The inter-rater reliabilities of the German version of the ADIS-IV-L in a patient sample (*n* = 237) were between .61 and .98 (Yule’s Y coefficient) for social anxiety disorder and between .73 and .98 (Yule’s Y coefficient) for specific phobia [56]. Retest-reliabilities across the groups of disorders were between .68 and .79 (Kappa coefficient) and between .67 and 1.0 (Yule’s coefficient) [56, 57]. In addition, the interview has demonstrated high validity [58, 59] as well as good acceptance in clinical practice and research settings [60].

#### Clinical severity rating

Based on the ADIS, the clinicians rated the severity of distress and interference for each disorder, on a scale ranging from 0 (not severe at all) to 8 (very severe). A diagnosis was considered clinically severe when the clinical severity rating was of 4 or above.

#### Interviewers, training procedure, and supervision

Interviewers were clinical psychologists, physicians, or advanced graduate students of clinical psychology. They underwent an intensive 40-hour training on mental disorders, their rating and the conduct of the interviews and subsequently attended supervision meetings every two weeks. The written protocol for each interview was proof read by supervisors (e.g., were all answers complete, and did the answers match the diagnosis assigned). Unclear cases were discussed, and if necessary a consensus diagnosis was given.

#### Avoidance behavior

Avoidance behavior at baseline and follow–up were measured by the German version of the ADIS-IV-L [53, 54]. In this structured interview, the interviewer read a list of 13 social situations (e.g., public speaking, talking to people in authority) and 17 specific situations (e.g., animals, heights) and asked the women to indicate if they ‘wanted to avoid’ each situation. If the answer was ‘yes’, the interviewer enquired about the degree of avoidance, which were rated on a scale of 0 (no avoidance) to 8 (very severe avoidance).

In the current study, the avoidance scores were summed separately for social and specific situations at the follow-up interview, with ratings anchored to ‘since the baseline interview’. The possible ranges are 0–104 for avoidance of social situations and 0–136 for avoidance of specific situations, respectively. These sum scores reflect circumscribed avoidance with higher scores indicating greater avoidance. In the current sample, the internal consistency (Cronbach’s α) was .76 for social situations and .70 for specific situations.

#### Beck Anxiety Inventory (BAI)

The Beck Anxiety Inventory (BAI) [61, 62] is a 21-item self-report inventory of the severity of anxiety symptoms (e.g., feeling hot, or fear of losing control). Participants were asked to rate how much each symptom bothered them ‘during the past week, including today’ on a 4-point-rating scale ranging from ‘not at all’ (0) to ‘severely–I could barely stand it’ (3). The possible range of the total score goes from 0 to 63 with scores between 0 and 21 indicating low anxiety, scores between 22 and 35 moderate anxiety, and scores of 36 and above indicating potentially concerning levels of anxiety. In the current sample, the BAI had good internal consistency (Cronbach’s α = .82).

### Definition of the groups

To study the moderating role of avoidance behavior on general anxiety over time in women with a social anxiety disorder and women with a specific phobia, two subgroups of the initial sample of 1,396 participants were composed based on the seven-day point prevalence at baseline of the ADIS-IV-L: The first group were 91 women with a baseline social anxiety disorder and the second group were 130 women with a baseline specific phobia. Comorbid cases were included in both groups. The datasets used in the present study are available in S1 and S2 Datasets. These group sizes resulted in more statistical power to detect substantial effects in the group of specific phobia relative to the group of social anxiety disorder [63]. Using the software program G*Power 3 [64], the power to detect a medium effect for the interaction (*f*^2^ = 0.15) with an alpha level of five percent is 99.2 percent for the model with 130 women (specific phobia group) and 95.5 percent for the model with 91 women (social anxiety disorder group). These power estimates drop drastically to 36.0% and 26.6% when the interaction effects turn out to be small (*f*^2^ = 0.02). To detect a small interaction effect with a power of .80, a sample size of 395 would be required. Thus, in addition to results of the significance tests, we rely on effect sizes as well. The average age at baseline was 21 years (*SD* = 1.8 to 1.9) for both groups.

### Statistical analyses

Ordinary least squares (OLS) regression analysis was used to examine the moderating effect of circumscribed avoidance behavior between baseline and follow-up on the relationship between general anxiety at baseline and 18 months later. The predictor and moderator variables were mean-centered prior to the analyses [65]. The centered predictor and moderator variables were then multiplied to form the interaction terms. Conducting hierarchical regression analysis, the simple effects of general anxiety at baseline (General anxiety baseline) and avoidance of situations between baseline and follow-up (Avoidance) were entered on the first step and the interaction term (General anxiety baseline × Avoidance) was entered on the second step. We used *f*^2^ as a measure of effect size for the interaction effect and considered effect sizes of .02, .15, and .35 as small, medium, and large, respectively [63].

## Results

### Preliminary analyses

Women with a baseline social anxiety disorder mostly feared public speaking (*Mdn* = 4.00, *M* = 4.04, *SD* = 2.12; possible range of anxiety severity: 0–8, with 0 = no anxiety and 8 = very severe anxiety). Less feared situations were talking to people in authority (*Mdn* = 2.00, *M* = 2.21, *SD* = 1.95), starting a conversation (*Mdn* = 2.00, *M* = 1.95, *SD* = 1.92), rejecting a senseless claim or asking someone to change his or her behavior (*Mdn* = 1.00, *M* = 1.77, *SD* = 1.65), and talking to strangers (*Mdn* = 1.00, *M* = 1.45, *SD* = 1.73). Women with a baseline specific phobia mostly feared animals (*Mdn* = 4.00, *M* = 3.99, *SD* = 2.60; possible range of anxiety severity: 0–8, with 0 = no anxiety and 8 = very severe anxiety). Less feared situations were heights (*Mdn* = 0.00, *M* = 1.66, *SD* = 2.20) and blood, injuries, and injections (*Mdn* = 0.58, *M* = 1.12, *SD* = 1.50). As expected, the means of clinical severity in women with a baseline social anxiety disorder and specific phobia were above the clinical cut-off of 4, providing further evidence that these individuals can be considered to be clinically severe (baseline social anxiety disorder: *Mdn* = 4.00, *M* = 4.24, *SD* = 1.30; baseline specific phobia: *Mdn* = 4.00, *M* = 4.32, *SD* = 1.25).

The co-occurring diagnoses at baseline and follow-up for the social anxiety disorder and specific phobia groups are given in Table 1. Among the 91 women with a social anxiety disorder diagnosis, 39 (42.9%) had comorbid diagnoses at baseline. The most frequent co-occurring diagnoses were another anxiety disorder, namely specific phobia, agoraphobia without panic disorder, and generalized anxiety disorder. Of the 91 women with baseline social anxiety disorder, 33 (36.3%) fulfilled the diagnostic criteria for social anxiety disorder also at follow-up. Among the 130 women with a specific phobia diagnosis, 32 (24.6%) had comorbid diagnoses at baseline. The most frequent co-occuring diagnoses were another anxiety disorder, namely social anxiety disorder, agoraphobia without panic disorder, and generalized anxiety disorder. Of the 130 women with baseline specific phobia, 52 (40.0%) fulfilled the diagnostic criteria for specific phobia also at follow-up.

**Table 1:** Diagnoses at baseline and follow-up for the social anxiety disorder and specific phobia groups.

Diagnoses	Baseline SAD (*n* = 91)				Baseline SP (*n* = 130)			
	Baseline		Follow-up		Baseline		Follow-up	
	*n*	%	*n*	%	*n*	%	*n*	%
Anxiety disorders								
PD without AGR	1	1.1	1	1.1	0	0.0	4	3.1
PD with AGR	2	2.2	2	2.2	1	0.8	2	1.5
AGR without PD	7	7.7	5	5.5	6	4.6	4	3.1
SAD	91	100	33	36.3	18	13.8	13	10.0
SP	18	19.8	19	20.9	130	100	52	40.0
GAD	5	5.5	5	5.5	4	3.1	7	5.4
OCD	3	3.3	4	4.4	2	1.5	2	1.5
PTSD	3	3.3	0	0.0	1	0.8	0	0.0
Affective disorders	3	3.3	7	7.7	3	2.3	6	4.6
Somatoform disorders	0	0.0	3	3.3	3	2.3	3	2.3
Substance use disorders	4	4.4	3	3.3	2	1.5	3	2.3
Eating disorders	4	4.4	5	5.5	1	0.8	3	2.3

*Note*. PD = panic disorder. AGR = agoraphobia. SAD = social anxiety disorder. SP = specific phobia. GAD = generalized anxiety disorder. OCD = obsessive-compulsive disorder. PTSD = posttraumatic stress disorder.

Product-moment correlations between baseline general anxiety and avoidance were .02 for the social anxiety disorder group and .09 for the specific phobia group. Descriptive statistics are given in Table 2. In the specific phobia group, the general anxiety measure at baseline was in average significantly higher than at follow-up, *t*(128) = 2.726, *p* = .007, Cohen’s *d* = 0.240, but not in the social anxiety disorder group, *t*(90) = 1.852, *p* = .067, Cohen’s *d* = 0.194.

**Table 2:** Means, standard deviations, and empirical ranges for the study variables for the social anxiety disorder and specific phobia groups.

Variable	Baseline SAD (*n* = 91)			Baseline SP (*n* = 130)		
	*M*	*SD*	Range	*M*	*SD*	*Range*
Avoidance	10.45	8.19	0–31	12.13	10.96	0–45
General anxiety baseline	8.09	6.70	0–28	6.07	5.64	0–28
General anxiety follow-up	6.82	6.73	0–31	4.75	5.77	0–28
Number of disorders	1.56	0.75	1–4	1.33	0.68	1–5

*Note*. SAD = social anxiety disorder. SP = specific phobia. Avoidance = Avoidance between baseline and follow-up. *M* = Mean, *SD* = Standard deviation. Number of disorders = Number of disorders total at baseline. Possible ranges: 0–104 for avoidance of social situations, 0–136 for avoidance of specific situations, 0–63 for general anxiety.

### Main analyses

The moderating effect of circumscribed avoidance behavior between baseline and follow-up on the relationship between general anxiety at baseline and general anxiety at follow-up was analyzed separately for women with social anxiety disorder and women with specific phobia. The results of these models are given in Table 3.

**Table 3:** Regression analyses for the social anxiety disorder and specific phobia groups testing whether the relationship between general anxiety at baseline and at follow-up is moderated by avoidance behavior between baseline and follow-up.

Predictor	Baseline SAD (*n* = 91)			Baseline SP (*n* = 130)		
	*b*	*SE*	*R*^2^	*b*	*SE*	*R*^2^
General anxiety baseline	0.491***	0.091	.840	0.492***	0.082	.881
Avoidance	0.151*	0.072	.118	0.047	0.038	.051
General anxiety baseline × Avoidance	0.012	0.009	.042	0.012*	0.005	.068
Intercept	6.801***	0.591		4.707***	0.419	

*Note*. SAD = social anxiety disorder. SP = specific phobia. General anxiety baseline and Avoidance were mean-centered prior to the analyses.

*p < .05.

***p < .001 (2-tailed).

#### Social anxiety disorder

Both simple effects were, as expected, positive and statistically significant for the social anxiety disorder group, revealing that the higher the general anxiety at baseline and the higher avoidance behavior the higher the level of general anxiety at follow-up. Contrary to our second hypothesis, the interaction effect was not statistically significant, indicating that the relationship between general anxiety at baseline and at follow-up was not moderated by the level of avoidance behavior. The total explained variance was 32.1%, and the variance explained by the two simple effects was 30.7%. The effect size *f*^2^ of the interaction was .019, indicating no substantial interaction effect.

#### Specific phobia

**Fig 1:**
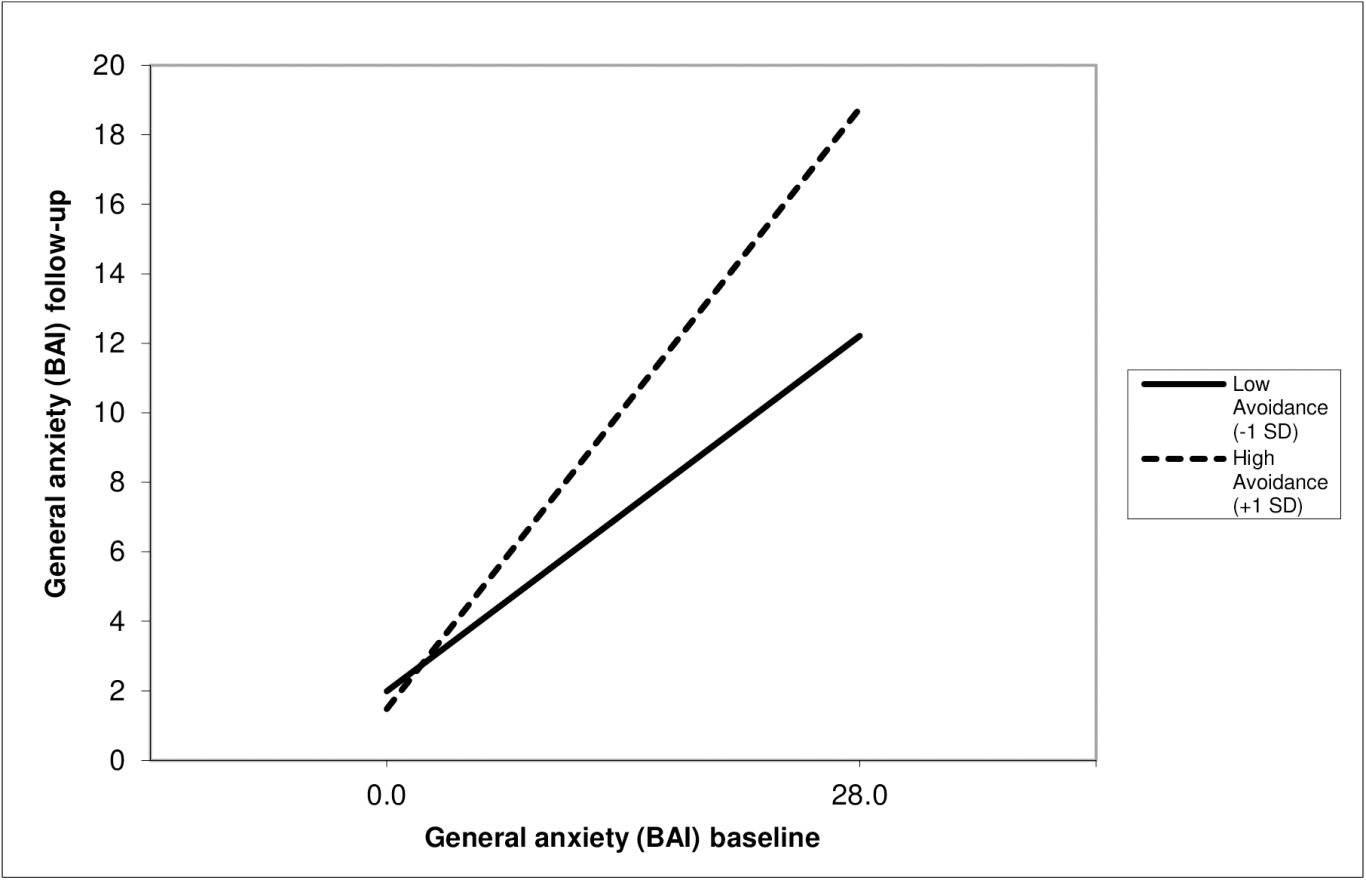
Regression lines of the specific phobia group showing the moderating effect of avoidance behavior of specific situations on the relationship between general anxiety at baseline and follow-up.

As expected, the simple effect of general anxiety at baseline was positive and statistically significant for the specific phobia group but the effect from avoidance was not significant, indicating that the higher the level of general anxiety at baseline the higher the level of general anxiety at follow-up. The interaction effect was, in line with the second hypothesis, statistically significant, indicating that the relationship between general anxiety at baseline and at follow-up was moderated by the level of avoidance behavior. The relationship between anxiety at baseline and follow-up for low avoidance, defined as the mean minus 1 *SD* (i.e., -10.96), and high avoidance, defined as the mean plus one *SD* (i.e., 10.96), is presented in Fig 1. As can be seen, in both, women with low avoidance and women with high avoidance general anxiety decreased, yet this decrease was substantially lower in women with low avoidance. Using the Johnson-Neyman [66] technique to identify regions of significance, the effect between general anxiety at baseline and follow-up was statistically significant for the whole range of avoidance behavior. This means that for any possible value of avoidance behavior the relationship between general anxiety at baseline and follow-up is statistically significant. The total explained variance was 34.5%, the variance explained by the two simple effects was 32.1%. The effect size of the interaction was .036, which–although small in size according to Cohen’s thumb of role–is substantially bigger than the interaction in the social anxiety disorder group.

In sum, these results suggest that avoidance behavior seems to have a more direct influence on general anxiety at follow-up for women with a social anxiety disorder. In contrast, in women with a specific phobia avoidance behavior seems to have a moderating effect on the relationship between general anxiety at baseline and follow-up.

### Expected change

A question of practical interest may be how much change we can expect in general anxiety from baseline to follow-up depending on the level of avoidance behavior between baseline and follow-up for a particular person. For instance, for a woman with a social anxiety disorder and a score of general anxiety at baseline of 5.0, the expected general anxiety score at follow-up is 4.7 if her avoidance score is 0 and 9.0 if her avoidance score is 30. For a woman with a specific phobia and a score of general anxiety at baseline of 5.0, the expected general anxiety score at follow-up is 3.9 if her avoidance score is 0 and 5.1 if her avoidance is 30.

### Additional analyses

We rerun the regression analyses controlling for a set of potential confounding variables. First we controlled for the number of other mental disorders at baseline for the social anxiety disorder and specific phobia group and found that the pattern did not change. Second, we controlled for whether participants received treatment because a few participants received treatment for personal or emotional problems in the intermediate interval (9 women or 9.9% with social anxiety disorder and 13 women or 10.0% with specific phobia). Again, controlling for possible treatment did not significantly alter the interaction effects or the simple effects. We note, however, that the inclusion of these control variables had a negative effect on the distribution of the residuals, violating the assumption of normally distributed residuals.

## Discussion

The current study aimed to examine the mechanism by which circumscribed avoidance behavior contributes to the maintenance of general anxiety in women with a baseline social anxiety disorder and a baseline specific phobia. In line with findings of studies investigating the effect of exposure-based treatments [32, 33, 35, 39], the current results support that low circumscribed avoidance behavior can lower general anxiety over time. However, the mechanism by which avoidance behavior did so was different for women with a DSM-IV specific phobia and women with a DSM-IV social anxiety disorder. As would be expected from cognitive theory [15] and overprediction of fear [18], avoidance behavior moderated the strength of the association between general anxiety at baseline and at follow-up in women with a specific phobia. As illustrated in the example, low avoidance had a buffering effect, whereas very high avoidance amplified the strength of the relationship between baseline and follow-up general anxiety. In contrast, in women with a diagnosed social anxiety disorder, circumscribed avoidance behavior was found to have a direct but no moderating effect on general anxiety at follow up.

There are several explanations for the different role avoidance behavior plays in explaining the maintenance of anxiety in specific phobia and social anxiety disorder. One explanation is that individuals with a specific phobia may be more successful in their avoidance of feared situations than individuals with a social anxiety disorder, since social situations (e.g., starting a conversation) are often more unavoidable to a certain degree than specific situations (e.g., heights). Another explanation is, that individuals with a specific phobia may experience more positive outcomes after approach behavior than individuals with a social anxiety disorder due to biases in information processing [67–69]. For instance, individuals with a social anxiety disorder may interpret ambiguous stimuli (e.g., frowns, signs of boredom) as indicators of disapproval, which increases their anxiety. Hence, avoidance behavior may, in line with the current results, more directly influence anxiety in specific phobia, whereas the effects of approach interfere with the disconfirmation of negative beliefs in social anxiety disorder. Moreover, avoidance behavior may be more subtle in social anxiety disorder than specific phobia, such as the reliance on ‘in-situation or subtle safety behaviors’ that contribute to the maintenance or exacerbation of anxiety [25, 26]. The ratings of avoidance employed herein did not cover such ‘in-situation or subtle safety behaviors’, resulting in lower sensitivity for measuring the effects of avoidance in social anxiety disorder.

The findings of this study add to the growing number of studies investigating the role of important moderating variables (e.g., attentional control, anxiety sensitivity) in anxiety and phobia research [70, 71]. In addition, our study supports the finding that young adolescents in the general population have a relatively high likelihood of remission for social anxiety disorder and specific phobia. In our study, 64% of the women with a baseline social anxiety disorder did not fulfill all criteria for a DSM-IV social anxiety disorder 18 months later. This finding is in line with results from other community-based prospective studies with remission rates for social anxiety disorder between 54 and 93 percent over a time interval of 1 to 4 years in 14- to 65-year olds [72, 73]. In the current study, 60% of the women with a baseline specific phobia did not fulfill all criteria for a DSM-IV specific phobia at follow-up. A similar rate was reported in another prospective community-based study, with 70% of the 14-to 17-year old adolescents showing remission of threshold and subthreshold specific phobia over a 19-month time interval [74]. Other major strengths of the present study include the longitudinal design, the standardized assessment of DSM-IV diagnoses of specific phobia and social anxiety disorder, and the large sample size of 221 women with a corresponding anxiety disorder. However, there are also a few limitations. First, the measure of avoidance was based on retrospective reports covering the period between the baseline and follow-up interview. Nonetheless, retrospective reports of anxiety symptoms have been shown to be fairly accurate in past studies [75, 76]. Second, there may be differences in the effect of the moderating role of avoidance behavior for singular social and specific situations, such as public speaking or animals. This could be addressed in further studies since our data did not enable a meaningful examination of single situations. Third, although we have made attempts to address issues of overlap of diagnoses in the two groups, it would be interesting to examine women with a pure social anxiety disorder relative to women with a pure specific phobia. However, the limited number of diagnosed women in our study did not allow us to examine this question. Finally, our sample consisted of a community sample of young women aged between 18 and 24 years only, all of whom lived in Germany. The results need to be verified for younger and older populations as well as males.

In conclusion, the current study showed that circumscribed avoidance behavior moderated the relationship between general anxiety at baseline and at follow-up in women with a baseline specific phobia, whereas in women with a baseline social anxiety disorder circumscribed avoidance behavior was directly associated with general anxiety at follow-up. The findings also highlight the crucial role that avoidance behavior plays in the maintenance of general anxiety in both women with a social anxiety disorder and women with a specific phobia.

## Supporting information

S1 Dataset Social anxiety disorder (*n* = 91). (XLSX)

S2 Dataset Specific phobia (*n* = 130). (XLSX)
